# Integrating Business and Medicine: A Scoping Review of Curricular Integration in U.S. Undergraduate Medical Education

**DOI:** 10.1007/s40670-026-02835-z

**Published:** 2026-07-27

**Authors:** Harrison Smith, Gemma Toogood, Grace Muncy, Meghan Underwood, John Calvin Bryant, Pallavi Khanna, Laura Himel

**Affiliations:** College of Medicine, University of Tennessee Health Science Center, 910 Madison Avenue, Memphis, TN 38163 USA

**Keywords:** Curriculum, Undergraduate medical education, Business, Education development, Physician leadership

## Abstract

**Purpose:**

A scoping review was conducted to evaluate business curriculum in U.S. undergraduate medical education (UME).

**Methods:**

A search (PRISMA-ScR) of research databases was conducted to identify studies evaluating U.S. allopathic medical school business curriculum. Data was synthesized qualitatively.

**Results:**

The 17 studies meeting inclusion criteria were highly heterogeneous. Reported qualitative outcomes demonstrated a theme recognizing the need for business education and a lack of consistent outcome measurement or rigorous methodology.

**Conclusion:**

An important gap exists regarding business curricula in UME and literature is limited. A consensus on core competencies is a necessary first step before educational development and evaluation.

**Supplementary Information:**

The online version contains supplementary material available at https://doi.org/10.1007/s40670-026-02835-z.

## Introduction

In 2002, only 26% of US medical schools offered a joint MD/MBA program. Today, this has grown to over 60% of US medical schools offering a joint MD/MBA degree [1]. Similarly, medical students have continued interest in business curriculum during medical school [2]. This trend also reflects the realities of modern medicine, in which increasing corporatization and evolving care models require physicians to have a stronger understanding of business management.

Integration of business education in undergraduate medical education (UME) equips future physicians with the skills and experience to transition to non-clinical healthcare roles in the new landscape of the complex healthcare system [3]. Programs such as joint MD/MBAs provide physicians with an overview of organizational management, finance, and conflict resolution, helping them navigate the business components of clinical and nonclinical medicine [4]. Business education provides physicians with the mobility to maximize their impact on healthcare by pursuing both clinical and nonclinical careers by further allowing physicians to have a role in healthcare management, leadership, and policy [5, 6]. Leadership education has become an important part of UME curricula, with a well-recognized role in the early development of physicians [7–9]. Although interest in incorporating business education into UME is rising and the demand for physicians to understand the business of medicine is well recognized, there remains a significant gap in both qualitative and quantitative research on how such curricula are integrated into UME [10–12].

This study aimed to demonstrate the gaps in currently available literature directly evaluating the current integration of business and UME curriculum, to identify potential spaces for changes in curriculum, and next steps in the integration of business education during undergraduate medical education.

## Methods

The review was conducted in accordance with the Preferred Reporting Items for Systematic Reviews and Meta-Analyses-Scoping Review (PRISMA-ScR). Scoping review strategy was utilized due to the heterogeneity seen in the initial literature review for both the types of studies conducted and the reported results.

### Search Strategy

A comprehensive search strategy was developed in collaboration with ChatGPT 4o (Pro Subscription, OpenAI) to identify studies evaluating business, entrepreneurship, or leadership education within undergraduate medical education curricula. The search included Medical Subject Headings (MeSH) and keyword terms selected by the authors and expanded using ChatGPT 4o to capture indexed and non-indexed terms. Then, this agent was utilized to format the search terms in accordance with each database’s advanced search syntax. PubMed, Embase, Scopus, and ERIC (EBSCOhost) were queried in February 2025. The full search strategy is attached in Supplementary Material A. To identify additional studies not captured by the search terms, the reference lists of included studies were manually back searched for additional studies.

### Inclusion Criteria

This review evaluated current literature on business curriculum in UME in the United States. Studies included were conducted at or commented on United States (regardless of publishing country) allopathic medical schools with business curricular interventions and dual degree programs. Business curriculum was defined as curricula including but not limited to healthcare economics, systems operations, monetary impact of insurance, and physician economic leadership. Only reports written in English were included.

### Exclusion Criteria

Studies on business education for residents, non-United States medical school programs, osteopathic schools, and other health degree (dental, nursing, physician’s assistant, etc.) programs were excluded. Curriculum interventions based solely on leadership development, innovation or entrepreneurship with an engineering focus, and interventions (e.g. residency, physician executive MBA, or courses for practicing physicians) were excluded. Only completed studies and articles were included.

### Study Selection

**Fig. 1:**
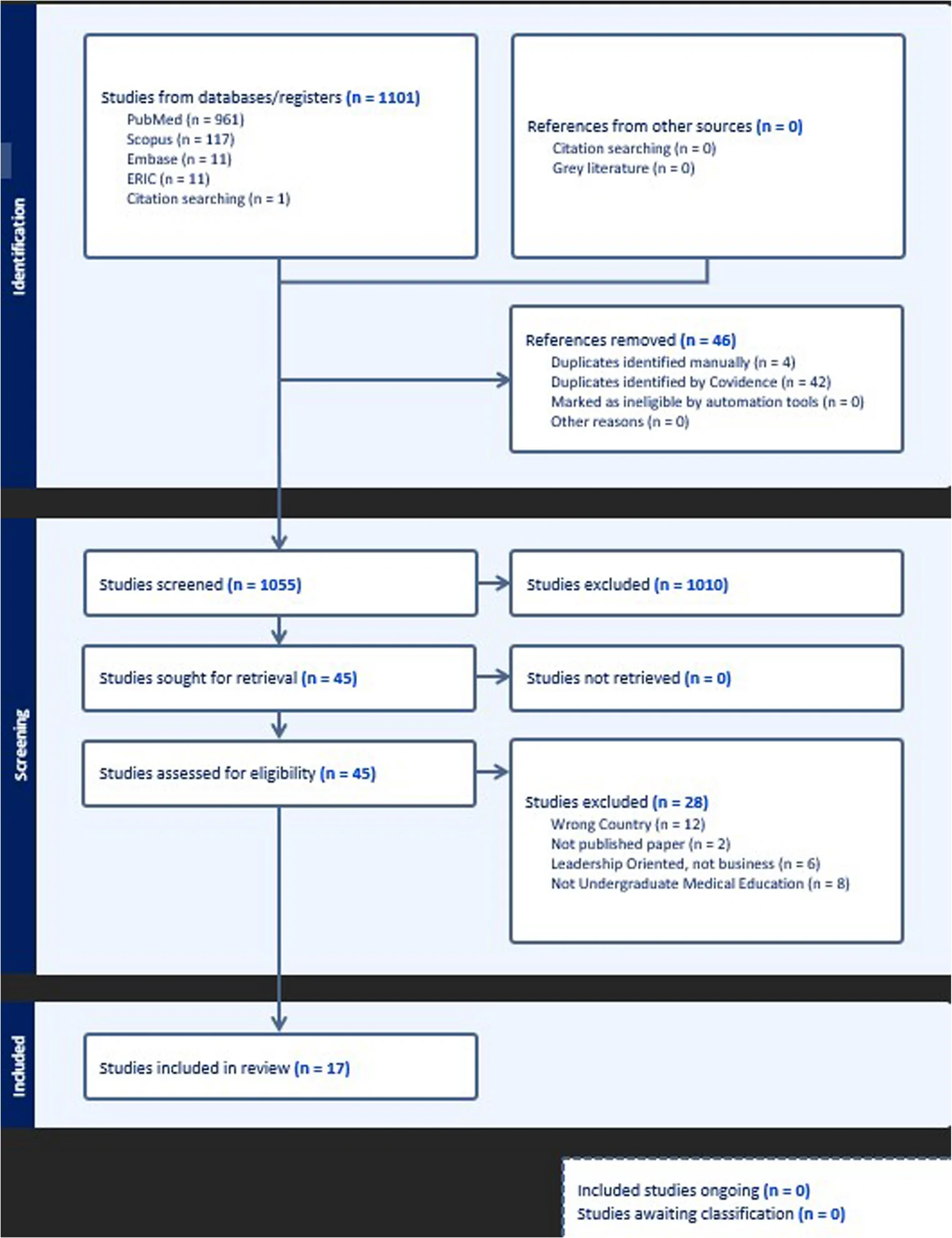
PRISMA flow diagram of included and excluded studies generated by Covidence

All identified records were uploaded to Covidence (Veritas Health Innovation, Melbourne, Australia) for screening and data extraction. Duplicates were removed automatically by Covidence. Titles and abstracts were screened by two independent reviewers, and identified conflicts were resolved by a third reviewer. The same process was followed for the full text screen. This process is summarized in a PRISMA flow diagram (Fig. 1). Inter-rater reliability was measured using Cohen’s Kappa calculated for full text review, as calculated by Covidence.

### Data Extraction and Presentation

Data was extracted by two reviewers using the data extraction tool developed by the reviewers in Covidence and imported into an Excel file. Extracted data included author details, study design, aim of the study, description of educational intervention, sample size, and study conclusions. Any disagreements between reviewers were resolved by a third reviewer. Data was synthesized qualitatively and semantic thematic analysis was completed. The findings from the included studies will be described narratively due to the heterogeneity of the studies.

The Critical Appraisal Skills Program (CASP) was utilized for risk of bias assessment [13]. The methodological quality of the articles included was assessed by two independent reviewers using the CASP checklist [13]. Discrepancies were resolved through discussion and consensus.

## Results

We identified 1,101 records for screening from database searches, of which 17 manuscripts published were identified as meeting our inclusion criteria (Table 1).

**Table 1:** Characteristics of included studies on business education in medical training [5, 6, 14–28].

Study	Country	Design	Aim	Intervention	Sample	Key Findings
Andrews 2016	USA	Cross-sectional survey	Explore medical students’ perspectives on fourth-year educational activities	Curricular needs assessment	376 students	Business skills not highly rated by students as important in fourth year. Provides student perspective on business knowledge prioritization.
Crites 2008	USA	Curriculum discussion	Build model leadership curriculum integrating leadership education across MD, MPH, and MBA programs	Curricular development	N/A	Developed integrated curriculum with MD, leadership, and MPH/MBA components. Three of five curricular interventions involve business-related activities including health systems management and strategic healthcare communications.
Goyal 2015	USA	Survey/Thematic analysis	Explore MD/MBA program structures, admissions, timelines, and coursework	Joint degree (MD/MBA)	65 programs	Wide variability in program structures (mostly 5 years). Topics include healthcare administration, medical ethics/law, and health economics. Number of MD/MBA programs increased over 19 years.
Hollis 2021	USA, Morocco	Survey/Thematic analysis	Investigate career aspirations, perceived skills, and enrollment reasons of MD/MBA students	Joint degree (MD/MBA)	67 students (14 programs)	Evaluation of career aspirations and acquired skills in MD/MBA programs. Survey analysis of student perspectives on dual degree benefits.
Hsu 2007	USA	Opinion/Commentary	Argue for business curriculum by examining current medical education landscape and past integration attempts	Commentary	N/A	Current undergraduate medical education does not prepare physicians for business-healthcare intersection challenges. Presents framework for incorporating business/management into medical curriculum.
Krupat 2017	USA	Survey/Thematic analysis	Examine MD/MBA program concerns regarding student attraction and impact on professional identity	Joint degree (MD/MBA)	34 students	MD/MBA students comparable to MD-only students in most ways. Examined impact of medicine and business culture exposure on professional identities, motivations, and career aspirations.
Larson 2003	USA	Survey/Thematic analysis	Measure changes in number and nature of MD/MBA program affiliations	Joint degree (MD/MBA)	33 programs, 329 students	Dramatic increase in students seeking MD/MBA and schools sponsoring programs. These graduates represent potential new physician leaders. Growth indicates rising cooperation between medical and business schools.
Lerman 2018	Saudi Arabia	Opinion/Literature review	Review current thinking on leader development in medicine, focusing on approaches, challenges, and opportunities	Literature synthesis	N/A	Business is key part of leadership development in medicine. Education can increase leadership competencies, but approaches differ. Ultimate goals depend on respective roles.
Myers 2017	USA	Opinion/Commentary	Encourage medical schools to partner with business faculty for ‘Management 101’ course	Curricular proposal	N/A	Proposes embedding management fundamentals into medical curriculum through business school partnerships. Advocates for continual business education beginning in medical school throughout career.
Niccum 2017	USA	Landscape review/Thematic analysis	Build model leadership curriculum integrating leadership education across degree programs	Curricular development	N/A	Developed integrated educational solution combining clinical care, healthcare leadership, and business/public health management. Early integration of leadership curriculum may benefit graduates and organizations sooner.
Patel 2014	USA, Panama	Survey/Thematic analysis	Evaluate graduates’ perceptions of MD/MBA training on career and professional development	Joint degree (MD/MBA)	148 respondents	MD/MBA graduates report mostly positive attitudes. Many pursue leadership and nonclinical roles later in careers. Large portion obtained dual degree during training.
Portney 2019	USA	Survey/Thematic analysis	Provide medical students didactic training and project-based learning for healthcare leadership	Optional curriculum	14 students	Effective community-based intervention. Students developed project and business proposals with nonprofit partners. Students continue as project leads to grow program.
Silverstein 2025	Canada	Qualitative interviews	Understand how STARS program impacted participants’ knowledge, skills, and career plans	Optional curriculum	27 students	Students gained resource stewardship knowledge, enhanced change management skills (leadership, advocacy, collaboration), and commitment to high-value care. Framework applicable to other curricular interventions.
Sklar 2018	USA	Opinion/Commentary	Examine thoughts on organized leadership training	Commentary	N/A	Skepticism about current healthcare leadership training programs. Emphasizes importance of effective, diverse, multidisciplinary-oriented leadership.
Stoller 2017	USA	Curriculum discussion	Provide roadmap for clinicians to expand leadership skills through a 5-month didactic/experiential curriculum	Optional curriculum	18 clinicians	Program aligns with expert opinion on ideal leadership training. Emphasizes medical school as starting point for leadership education. Advocates for business development programs similar to other sectors.
Sweigart 2016	USA, Cayman Islands, Colombia	Prospective cohort	Evaluate Health Innovations Scholars Program (HISP), a 5-week summer program for preclinical students	Summer program	6 students	Significant improvement in all three subject areas (leadership, quality/safety, operational efficiency). Successfully advanced preclinical students’ ability to lead clinical systems improvement.
Varkey 2009	USA	Mixed methods	Elicit perspectives on knowledge and competencies needed in undergraduate leadership curriculum	Curricular needs assessment	22 faculty, 21 students	Framework for leadership curriculum focus areas. Business aspects (coding/billing, investment) recognized as important but take backseat to other leadership aspects. Students lack confidence despite recognizing importance.

^1^*MD* Doctor of Medicine, *MBA* Master of Business Administration, *MPH* Master of Public Health, *USA* United States of America, *N/A* not applicable, *STARS* Students and Trainees Advocating for Resource Stewardship, *HISP* Health Innovations Scholars Program

The full characteristics and raw data of the 17 included manuscripts are stated in Supplementary Material B. Moderate inter-rater reliability was seen between reviewers (к=0.55).

The 17 reports were published from 2003 to 2025, and there was a slight increase in publication trends, with 11 studies published since 2016 compared to 6 studies prior to 2016. Fifteen of the selected papers were from the United States, and 3 of these 15 papers were published in multiple countries (Cayman Islands, Colombia, Panama, and Morocco). 1 paper was from Saudi Arabia, and 1 paper was from Canada [5, 6, 20, 27]. The largest group of selected manuscripts were analyzed surveys, with heterogeneous publication types.

### Demographic Characteristics

The population characteristics of the papers were overall heterogeneous; however, some manuscripts had no selected population. For available data, average ages were from 27.9 in medical school cohorts to 53.4 in post-medical graduate cohorts [5, 6, 14, 26]. No specific trend in male or female predominance was noted. Seven manuscripts assessed curriculum interventions or opinions on curricular changes for current undergraduate medical students through surveys [5, 6, 14, 16, 18, 19, 23]. Within these seven manuscripts, selected responses exhibited further variation by background or school type (military medical graduates, students with prior consulting experience) or by year in school (ranging from pre-clinical to clinical students). Other selected populations include administrators at MD/MBA programs, healthcare management MBA graduates, MBA, and MD faculty.

### Type and Structure of Curricular Interventions

Across the included studies, business-related educational interventions demonstrated substantial variability in structure. Three primary curricular models were identified.

First, dual-degree programs represented the most comprehensive educational structure. Five studies described formal five-year MD-MBA or equivalent dual-degree pathways, in which students completed an additional year of training focused on business management, healthcare finance, or organizational leadership [5, 6, 16, 18, 19]. These programs are typically longitudinal and designed to provide in-depth exposure to administrative and economic aspects of healthcare delivery.

Second, standalone leadership or business-focused curricular experiences were described in five manuscripts [21, 23, 24, 26, 27]. These interventions did not confer an additional academic degree and were generally structured as elective modules, workshops, or certificate-style programs. Their duration varied considerably, ranging from short intensive experiences to longitudinal leadership development tracks embedded within existing institutional programming [15, 27].

Third, integrated curricular models were identified in two studies. One described a structured Physician Leadership Development Program that extended the duration of undergraduate medical education to five years by incorporating formal instruction in leadership, business management, and public health principles alongside traditional medical training [15]. Another study reported integration of business-adjacent competencies through Students and Trainees Advocating for Resource Stewardship (STARS) team-based learning and structured leadership opportunities embedded within the standard medical education curriculum without extending program length [24].

Taken together, these findings highlight the absence of a standardized structural approach to business education within undergraduate medical training, with programs differing markedly in duration, depth of exposure, and degree of formal credentialing.

### Stated Aims of Manuscripts

The stated aims of the reviewed publications had notable variability. Stated aims included exploring the effects of already implemented curriculum (MD/MBA programs or shorter curricular additions to pre-existing UME), calls for expansion of business curriculum in the UME curriculum through opinion pieces or analysis of current student/faculty responses, and evaluations of previous graduates’ perception of the utility or effectiveness of already implemented curriculum [5, 17, 18, 27, 28]. Three of the screened publications utilized newly implemented curricular interventions [23, 26, 27]. Four were personal commentary pieces [17, 20, 21, 25]. In terms of the papers exploring the implemented programs, the actual length of the programs had a wide variance. Terminal degree programs ranged from one year to six years, while the curricular interventions ranged from five weeks to one year [15, 16, 18].

### Analysis Characteristics

All 17 of the publications’ measures were either qualitative or thematic analysis, with inconsistent measurements of the results. Generally, outcomes were also qualitative. Themes included opinions on whether and how business curricula should be implemented within the medical curriculum, how current strategies have affected either physicians’ career outcomes or processes within the healthcare field, and leadership qualities that may be seen in participants of these programs.

### Key Findings

Reviews, commentaries and curriculum development focused manuscripts suggest that combining clinical care with healthcare leadership and business management early in training subjectively benefits physicians’ self-reported knowledge of the business and leadership of medicine with limited objective measurement of career or health system outcome benefit. Business knowledge was identified as a key component of leadership development, and formal partnerships with business faculty or programs are potential strategies. However, there was a continued discussion of skepticism on how to integrate these ideas without taking away from medical education with an agreement that these topics are important overall.

Surveys and thematic analyses showed an increase in the number of MD/MBA programs and student enrollment over the last two decades [16, 19]. Graduates of these programs generally report positive attitudes and often transition into leadership and non-clinical roles later in their career [5, 6, 18]. Student perspectives also have shown recognition of the importance of business education with a lack of confidence on these topics [28]. However, Andrews 2016 showed business skills were not highly rated by fourth year students [14].

Cohort studies and program evaluations showed short-term, intensive interventions demonstrated improvements in students’ understanding of leadership, quality, and efficiency, and optional curricula were also seen to have enhanced students’ management skills and resource stewardship [23, 24, 27].

### Quality Assessment and Inter-rater Reliability

The overall quality of the studies was variable (Supplementary Material C). While most of the articles had sound methodology in research question or design, limited sample sizes or a business-adjacent focus limited applicability. The majority of articles clearly articulated their research aims (CASP Question 1) and utilized a research design appropriate to address those aims (CASP Question 3). However, weaknesses were prevalent in reporting. Six studies provided insufficient detail (or it was not applicable) on their recruitment strategy (CASP Question 4), making it difficult to assess the risk of selection bias [15, 17, 20–22, 25]. Furthermore, data collection methods (CASP Question 5) were often vaguely described [15, 17, 20, 21, 25, 26]. Rigor in data analysis (CASP Question 8) was another common limitation with seven papers lacking a clear description of how data were handled or how themes were derived [15–17, 20, 21, 25, 26]. While most studies presented a clear statement of findings (CASP Question 9), the methodological limitations reduce the overall confidence in and transferability of the collective results.

Cohen’s Kappa was calculated to be 0.54, indicating moderate inter-rater reliability. The majority of disagreements were from classification of what entailed leadership versus business curriculum due to the frequent overlap in the topics of the available literature.

### Evidence Gaps Identified

Semantic thematic synthesis of the included literature highlighted several recurring gaps in the current evidence base. These included the absence of standardized competency frameworks to guide curricular development, limited use of rigorous evaluation methodologies, and a lack of consensus regarding the optimal timing and structure of business education within UME. Additionally, studies demonstrated an unclear balance between the desired breadth of curricular exposure and the practical constraints of an already crowded medical curriculum, seen in the variable lengths and topics of curricula. Collectively, these gaps represent important targets for future educational research and curriculum innovation.

## Discussion

Our findings reveal that the integration of business-related topics into UME in the United States represents an emerging but currently underdeveloped field of inquiry and practice. The body of literature is characterized by a limited volume of manuscripts that are markedly heterogeneous in their curricular focus, pedagogical approach, and level of integration. Programs and discussions cover a wide spectrum of topics, and implementation is variable. The predominance of lower-evidence level educational outcome metrics reflects the methodological immaturity of this emerging curricular domain.

As illustrated by this review, the state of business education in medicine stands in stark contrast to the escalating complexity of the modern healthcare environment. Moving beyond purely biologic and practical clinical foundations, adding medical business for UME has become an increasingly important tenet of training the future physician [21, 29]. Today, physicians are not solely clinicians, but they are actors within complex socio-ecological and economic systems where clinical decisions are linked to organizational, financial, and policy considerations [30]. Despite growth in MD/MBA programs, based on this review’s findings there is still a lack of widespread adoption of UME business education and limited, evidence-based evaluations of existing curricula, UME there is an overall trend towards focusing on individual skill acquisition for physicians looking to move into leadership roles within the healthcare financial system.

### The Imperative for Business Knowledge in 21st -Century Medicine

There is a powerful and growing consensus that lasting improvements in population health necessitate the integration of clinical care with the principles of public health, an understanding of healthcare delivery systems, and the physician’s active role within this system [31–33]. Similarly, integrated curricula that combine the sciences, public health, and systems-based learning have become a central feature in modern medical school [34–36]. Achieving these aims is not solely a clinical challenge, but it is a system, business, and leadership challenge. Physicians must be equipped to function as stewards of limited resources, lead interprofessional teams, and advocate for systemic change to improve individual and population health. The lack of time and space within an already crowded curriculum, along with the absence of a clear consensus on what is essential content and effective pedagogy exacerbates the challenge of adding business topics to UME. The lack of stringent, published curricular evaluations shown by our review is a potential feedback loop of limited evidence, creating limited change. Although medical schools and physicians recognize the importance of these new curriculum needs, without a solid foundation of evidence, hesitancy for true integration will likely continue.

### Competency Based Medicine Opportunities

New curriculum changes and needs have been highlighted since 1999 with the introduction of the six Core Competencies by the ACGME for residency, which has profoundly influenced UME [37, 38]. This shift has changed question from “Is there time and space to add a business course?” to “How can we design learning experiences that ensure our graduates are competent in navigating the complex systems of healthcare delivery?” Leveraging the ACGME’s established six core competencies—particularly Systems-Based Practice (SBP), which requires physicians to demonstrate “awareness and responsiveness to the larger context and system of health care”—offers a strong foundation for developing future curricula [39]. Applying these principles to UME through business curricula is a natural extension of existing expectations. A recurrent theme within the searched manuscripts was the focus on the individual physician’s change in career goals or specifically acquired skills from these programs. Although fundamental business skills are important in building a foundation of knowledge to approach the complex financial healthcare system, integrating topics such as broader healthcare economics finance or quality improvement will be vital in preparing competent physicians who meet established ACGME standards from the outset of their medical education.

### Limitations

This review is limited by the overall low number of research reports currently in the literature. The reliance on qualitative outcomes and the absence of a unified curricular foundation make it difficult to determine the short or long-term efficacy of these programs on student performance, career outcomes, or integration of business knowledge within the physician community. The lack of a unified curricular foundational set also limits conclusions, as different approaches have provided different results, and as most described curricular interventions were elective, participation may have been subject to self-selection bias, potentially contributing to more favorable perceptions of curricular integration. This heterogeneity was reflected in the moderate inter-rater reliability seen between reviewers. Search results may also have been limited by the classification of studies under business-specific headings, which may not have been covered under our medically focused search.

## Conclusion

The scarcity of information on business-specific curricula within UME represents an important gap in medical education, and the absence of established guidelines for what should be taught poses an additional challenge for future curriculum development. Consensus on the topics to be taught within a potential competency-based curriculum may represent an important preliminary step before attempts at educational development can be made. The studies included in this review suggest that the vast majority of the available literature falls at the lowest levels of the evidence pyramid. What research does exist focuses on physician career development rather than systems-based improvements. Establishing consensus on core competencies would enable standardized curriculum development, facilitate comparison across institutions, and permit rigorous evaluation of educational outcomes for business education in medicine.

## Supplementary Information

Below is the link to the electronic supplementary material.

Supplementary Material 1

Supplementary Material 2

Supplementary Material 3

## Data Availability

All data generated or analyzed during this study are included in this published article and its supplementary information files.

## Declarations

### Disclaimers

The authors have none to declare.
