# Supplementary material for: Integrating Business and Medicine: A Scoping Review of Curricular Integration in U.S. Undergraduate Medical Education
Source: Med Sci Educ. 2026 Jul 27;36(6):3239–47. doi: 10.1007/s40670-026-02835-z (PMC13759940; doi:10.1007/s40670-026-02835-z)
Supplement: Supplementary file 1 [file 40670_2026_2835_MOESM1_ESM.docx]

**Appendix A: Search strategy**

Search conducted on 2/19/2025 in PubMed.

| **#** | **Query** | **Results from 2/19/2025** |
| --- | --- | --- |
| 1 | ("Education, Medical"[MeSH] OR "Education, Medical, Undergraduate"[MeSH] OR "Curriculum"[MeSH] OR "Schools, Medical"[MeSH]) | 272,550 |
| 2 | ("Commerce/education"[MeSH] OR "Entrepreneurship"[MeSH] OR "Leadership"[MeSH]) | 53,435 |
| 3 | ("Program Evaluation"[MeSH] OR "Educational Measurement"[MeSH] OR "Professional Competence"[MeSH] OR "Clinical Competence"[MeSH]) | 252,997 |
| 4 | ("business education"[Title/Abstract] OR "management training"[Title/Abstract] OR "MBA"[Title/Abstract] OR "entrepreneurship education"[Title/Abstract]) | 5,601 |
| 5 | ("medical students"[Title/Abstract] OR "medical curriculum"[Title/Abstract]) | 57,500 |
| 6 | ("program effectiveness"[Title/Abstract] OR "knowledge assessment"[Title/Abstract] OR "competency evaluation"[Title/Abstract]) | 3,940 |
| 7 | ("Education, Pharmacy"[MeSH] OR "Pharmacy"[MeSH] OR "Education, Nursing"[MeSH] OR "Nursing"[MeSH]) | 345,927 |
| 8 | ("pharmacy"[Title/Abstract] OR "pharmacist"[Title/Abstract] OR "nursing"[Title/Abstract] OR "nurse"[Title/Abstract]) | 511,996 |
| 9 | ((english)) | 35,277,959 |
| 10 | (#1 AND #2 AND #3) OR #4 AND #5 AND #6 | 1,383 |
| 11 | #10 NOT #7 | 1,039 |
| 12 | #11 NOT #8 | 995 |
| 13 | #12 AND #9 | 972 |

**Embase**

**Date Searched:** 2/19/2025

**N=** 7

**Search Terms:**

('medical education'/exp OR 'undergraduate medical education'/exp OR 'curriculum'/exp OR 'medical school'/exp)

AND

('commerce education'/exp OR 'entrepreneurship'/exp OR 'leadership'/exp)

AND

('program evaluation'/exp OR 'educational measurement'/exp OR 'professional competence'/exp OR 'clinical competence'/exp)

OR

('business education':ti,ab OR 'management training':ti,ab OR 'MBA':ti,ab OR 'entrepreneurship education':ti,ab)

AND

('medical students':ti,ab OR 'medical curriculum':ti,ab)

AND

('program effectiveness':ti,ab OR 'knowledge assessment':ti,ab OR 'competency evaluation':ti,ab)

NOT

('pharmacy education'/exp OR 'pharmacy'/exp OR 'nursing education'/exp OR 'nursing'/exp)

NOT

('pharmacy':ti,ab OR 'pharmacist':ti,ab OR 'nursing':ti,ab OR 'nurse':ti,ab)

**Scopus**

**Date Searched:** 02/19/2025

**N=** 117

**Search Terms:**

( TITLE-ABS-KEY ( "medical education" OR "undergraduate medical education" OR "medical school curriculum" OR "medical school program" ) AND TITLE-ABS-KEY ( "business education" OR "commerce education" OR "entrepreneurship education" OR "leadership training" OR "management training" OR "MBA program" ) AND TITLE-ABS-KEY ( "program evaluation" OR "educational measurement" OR "competency-based education" OR "knowledge assessment" OR "professional competence" ) AND NOT TITLE-ABS-KEY ( "pharmacy education" OR "nursing education" OR "pharmacy students" OR "nursing students" OR "nursing curriculum" OR "pharmacist training" OR "nursing program" ) )

**ERIC**

**Date Searched:** 02/21/2025

**N=** 10

**Search Terms:**

(("medical education" OR "undergraduate medical education" OR "medical school curriculum" OR "medical school program")

AND

("business education" OR "commerce education" OR "entrepreneurship education" OR "leadership training" OR "management training" OR "MBA program")

AND

("program evaluation" OR "educational measurement" OR "competency-based education" OR "knowledge assessment" OR "professional competence"))

NOT

("pharmacy education" OR "nursing education" OR "pharmacy students" OR "nursing students" OR "nursing curriculum" OR "pharmacist training" OR "nursing program")
